# Usage and cost-effectiveness of elective oocyte freezing: a retrospective observational study

**DOI:** 10.1186/s12958-022-00996-1

**Published:** 2022-08-16

**Authors:** Ih-Jane Yang, Ming-Yih Wu, Kuang-Han Chao, Shin-Yi Wei, Yi-Yi Tsai, Ting-Chi Huang, Mei-Jou Chen, Shee-Uan Chen

**Affiliations:** 1 Department of Obstetrics and Gynecology, National Taiwan University Hospital, Number 8, Chung Shan South Road, Taipei City, 100 Taiwan; 2 Department of Obstetrics and Gynecology, National Taiwan University Hospital, Yunlin Branch, Yunlin County, 640 Taiwan; 3 Graduate Institute of Clinical Medicine, College of Medicine, National Taiwan University, Taipei City, 100 Taiwan; 4 Department of Obstetrics and Gynecology, National Taiwan University Hospital, Hsin-Chu Branch, Hsinchu City, 302 Taiwan; 5 Livia Shangyu Wan Chair Professor of Obstetrics and Gynecology, College of Medicine, National Taiwan University, Taipei City, 100 Taiwan

**Keywords:** Elective oocyte cryopreservation, Social oocyte freezing, Fertility preservation, Usage rate, Cost-effectiveness analysis

## Abstract

**Background:**

The previous model-based cost-effectiveness analyses regarding elective oocyte cryopreservation remained debatable, while the usage rate may influence the cost per live birth. The aim of this study is to disclose the usage and cost-effectiveness of the planned cryopreserved oocytes after oocyte thawing in real-world situations.

**Methods:**

This was a retrospective single-center observational study. Women who electively cryopreserved oocytes and returned to thaw the oocytes were categorized as thawed group. The oocytes were fertilized at our center and the sperm samples for each individual was retrieved from their respective husbands. Clinical outcomes were traced and the cumulative live birth rate per thawed case was calculated. The costs from oocyte freezing cycles to oocyte thawing, and embryo transfer cycles were accordingly estimated. The cumulative cost per live birth was defined by the cumulative cost divided by the live births per thawed case.

**Results:**

We recruited 645 women with 840 oocyte retrieval cycles for elective oocyte freezing from November 2002 to December 2020. The overall usage rate was 8.4% (54/645). After the storage duration exceeded ten years, the probabilities of thawing oocytes were 10.6%, 26.6%, and 12.7% from women who cryopreserved their oocytes at the age ≤ 35 years, 36–39 years, and ≥ 40 years, respectively (*P* = 0.304). Among women who thawed their oocytes, 31.5% (17/54) of women achieved at least one live birth. For the age groups of ≤ 35 years, 36–39 years, and ≥ 40 years, the cumulative live birth rates per thawed case were 63.6%, 42.3%, and 17.6%, respectively (*P* = 0.045), and the cumulative costs for one live birth were $11,704, $17,189, and $35,642, respectively (*P* < 0.001).

**Conclusions:**

The overall usage rate was 8.4% in our cohort. The cumulative live birth rate was greatest in the youngest group and the cumulative cost per live birth was highest in the oldest group, which was threefold greater than that in the group aged ≤ 35 years. The findings added to the limited evidence of the usage rate in real-world situations, which could hopefully aid future analysis and decision-making in public health policy and for women willing to preserve fertility.

**Trial registration:**

None.

**Supplementary Information:**

The online version contains supplementary material available at 10.1186/s12958-022-00996-1.

## Background

Planned oocyte cryopreservation, also referred to as *social/elective oocyte freezing*, is a strategy for women to use who wish to preserve their oocytes from age-related fertility loss [1, 2]. Fecundability significantly declines in women after 35 years of age [3, 4]. Lack of a suitable partner or incomplete self-accomplishment are the most frequent reasons prompting reproductive-aged women to defer childbearing [5, 6]. The idea of fertility preservation was primarily introduced to cancer patients, given that cancer treatment delayed the timing of conception and damaged the ovarian reserve [7]. The experimental label was further removed when the American Society for Reproductive Medicine and the European Society for Human Reproduction and Embryology (ESHRE) proposed that planned oocyte cryopreservation for age-related fertility loss is ethically permissible after individuals undergo a thorough consultation and have a reasonable expectation of costs, risks, and the likelihood of success [1, 2].

Investigators in the previous studies [8–11], which were mostly model-based, concluded that the results from cost-effectiveness analyses of social oocyte freezing remained controversial. The input variables that were used for the models usually included women’s age at oocyte freezing, live birth rates, costs for each procedure, and duration of storage years. However, only a fraction of the women would return to use the cryopreserved oocytes. Hence, the usage rate in the real world should also be considered. To date, although the number of women who choose to undergo fertility preservation has been gradually increasing, the evidence regarding the usage rate is scarce. Previous studies reported the usage rate of 12.1% and 15% for elective oocyte cryopreservation from Spain and Sweden, respectively [12, 13]. Another study in the United States [14], which followed up a cohort after 10–15 years of storage, revealed that 38.1% of patients thawed their oocytes. Additionally, studies also indicated that among women who used their oocytes, more than one-third elected sperm donation [14, 15]. In Taiwan, gamete cryopreservation is available to unmarried individuals for medical and nonmedical reasons. However, according to the Assisted Reproduction Act by the Ministry of Health and Welfare of Taiwan [16], in vitro fertilization (IVF) is exclusively provided to married heterosexual couples. The aim of our study was to assess the usage of planned cryopreserved oocytes and the cost-effectiveness after oocyte thawing.

## Methods

### Study design and population

This retrospective observational study was conducted at a medical center in Taipei, Taiwan. Women were included who underwent at least one oocyte retrieval cycle and oocyte freezing for social reasons between November 2002 and December 2020. Women were excluded from the study who cryopreserved oocytes because of medical reasons (e.g., women potentially receiving gonadotoxic treatment for malignant or nonmalignant diseases), male sex-related factors (e.g., men who could not provide semen or who had no motile sperm on the day of oocyte retrieval), or religious reasons (i.e., couples whose religious beliefs forbade embryo freezing).

### Ethical approval

This study was approved by the Institutional Review Board (IRB) of National Taiwan University Hospital (Taipei City, Taiwan; serial number 202104095RINB). The IRB waived the requirement for informed consent owing to the retrospective nature of the study.

### Controlled ovarian stimulation protocol and oocyte retrieval

The protocols for ovarian stimulation included the gonadotropin-releasing hormone (GnRH) antagonist protocol, progestin-primed ovarian stimulation (PPOS) protocol, short GnRH agonist protocol, and long GnRH agonist protocol. The details of each protocol have been previously described [17–20]. Levels of serum follicular-stimulating hormone, luteinizing hormone, and estradiol were measured on day 2 or day 3 of the menstrual cycle. The hormone levels and follicle sizes were followed up on stimulation day 5 or day 6 and the subsequent days. Final maturation of oocytes was triggered if the size of two follicles reached 18 mm and the estradiol level reached approximately 150 pg/mL per follicle. Oocyte retrieval was arranged 34–36 h post-triggering.

### Oocyte denudation and oocyte freezing

Oocytes were denuded after retrieval, and the maturation status of the oocytes was observed. Oocytes were frozen 2 h after oocyte retrieval. All metaphase II (MII) oocytes and metaphase I (MI) oocytes were frozen. The other immature oocytes were frozen after a discussion with the patient. The methods of oocyte freezing in our study included slow freezing and vitrification. Existing literature describes the detailed procedures for both methods [21–23]. The automated Kryo 10 series III biological vertical freezer (Planer Products, Ltd., Sunbury-on-Thames, United Kingdom) was used for the programmed cooling rate in the slow freezing method. In 2008, vitrification was introduced for oocyte cryopreservation in our laboratory. The Kitazato vitrification kit (Kitazato Supply Co., Tokyo, Japan) was used for vitrification [23]. The frozen oocytes were stored in liquid nitrogen tanks until the patients returned.

### Oocyte thawing

The method for thawing oocytes from the slow freezing method is described as follows [21, 22]. The straw was placed at 20ºC for 30 s, followed by soaking it in a 30ºC water bath for 40 s. The straw was incubated in 1.0 mol/L 1,2-propanediol (PROH) and 0.3 mol/L sucrose solution for 5 min, followed by incubation in a 0.5 mol/L PROH and 0.3 mol/L sucrose solution for 5 min. Subsequent incubation was carried out with 0.3 mol/L sucrose solution for 10 min. The final step of dilution was completed in phosphate-buffered saline solution with 20% maternal serum for 10 min at 20ºC and for another 10 min at 37ºC. The oocytes were placed in the human tubal fluid culture medium and were incubated until insemination. For vitrified oocytes, the Kitazato thawing kit (Kitazato Supply Co.) was used. The Cryotop vitrification device (Kitazato Supply Co.) was directly placed into the thawing solution and was kept immobile for 1 min. After the oocytes detached from the Cryotop, the oocytes were treated with the diluent solution for 3 min and washing solution for 5 min. They were then moved to the basic solution within 1 min at 20ºC. The oocytes were incubated in the human tubal fluid culture medium at 37ºC before insemination.

### Insemination, embryo culture, and transfer

Fertilization was conducted by using the matured oocytes that survived after 3 h of incubation. The method of fertilization used was intracytoplasmic sperm injection (ICSI), the details of which have been described in previous studies [22, 24, 25]. The first observation of the embryos was conducted 16–18 h after ICSI. Identification of two pronuclei (2PN) and a second polar body was defined as *normally fertilized*. A sequential culture medium was used for the embryo culture. The number and timing of embryo transfer were discussed with the patients based on the quality of the embryos. The remaining embryos were cultured and vitrified if blastocyst formation was observed.

### Endometrial preparation protocol for embryo transfer

Artificial hormone cycles were induced for endometrial preparation. Estrogen supplementation (Estrade, 2 mg/tab; Synmosa Biopharma Corp., Taipei City, Taiwan) was initiated on menstrual day 3 at a lower dose. The dose was increased to simulate the proliferative phase of the natural endometrium. Levels of serum hormone levels (i.e., luteinizing hormone, estradiol, and progesterone) were monitored and ultrasound was conducted on menstrual days 12 to 14. When the endometrium thickness reached 8–10 mm with a normal serum progesterone level (< 1.0 ng/mL), vaginal progesterone gel (Crinone; Fleet Laboratories Ltd., Watford, UK) was prescribed.

### Data collection and analysis

All demographic data and outcomes were collected by reviewing the patients’ medical charts. The baseline characteristics measured at the time of oocyte freezing included age, relationship, and body mass index. Ovarian conditions such as ovarian diseases, previous ovarian surgeries, and the Bologna criteria were also evaluated to determine whether a woman participant was a poor responder [26]. The ovarian stimulation protocols, medications, the number of retrieved oocytes and frozen oocytes, and the methods for oocyte freezing were reviewed. The disposition of the cryopreserved oocytes was followed up and was defined as *thawed*, *transported* (i.e., moving the oocytes to other infertility centers or countries), *discarded*, and *preserved*. If the participant thawed oocytes at our laboratory, the age at which the oocytes were thawed, the status of the thawed and survived oocytes, the number of normal fertilized and transferred embryos, and pregnancy outcomes were tracked. The costs of the oocyte freezing cycle and oocyte thawing cycle were estimated, which included the costs for medications, laboratory investigations, ultrasonographic examinations, procedures, and annual preservation fees.

The cases were stratified into three groups, based on the age at first oocyte freezing: ≤ 35 years, 36–39 years, and ≥ 40 years. The primary outcome was the cumulative cost per live birth from the thawed cases. The secondary outcomes were usage rate, oocyte survival rate, and live birth rate. *Oocyte survival rate* was defined as the number of survived oocytes divided by the number of thawed oocytes. *Clinical pregnancy* was defined as the detection of a gestational sac during ultrasound. *Ongoing pregnancy* was defined as a pregnancy in which at least one fetal heartbeat that continued after 12 weeks of gestational age. Pregnancy outcomes were confirmed as a *miscarriage* if the fetal loss occurred before 24 weeks or as a *delivery* if the woman gave birth to a child after 24 weeks. The effectiveness measure was the live births per thawed case. *Cumulative cost per live birth* was defined as the cumulative cost divided by the live births per thawed case.

### Statistical analysis

All data, which were not normally distributed, were determined for normality by using the Shapiro–Wilk test. The descriptive data were presented as the median and interquartile range (IQR) for continuous variables and as percentages for categorical variables. Nonparametric continuous variables were compared by using the Kruskal–Wallis test. Categorical variables were analyzed using the Chi-squared test. Šidák correction was conducted to adjust for multiple comparisons. Logistic regression was conducted for modeling and adjustment of covariates to evaluate the probability of live births. The cumulative incidence curve was presented, and the log-rank test was conducted to compare the probability of usage among different groups after several years of storage. A *P*-value less than 0.05 was statistically significant.

## Results

### Demographic data

A total of 645 women with 840 oocyte freezing cycles were included. The trend of the social oocyte freezing cycle per year in our center gradually grew from 2002 to 2020 and significantly increased in the past 10 years (e.g., 2010–2020) (Supplementary Fig. 1). The mean age at the first oocyte retrieval cycle was 37.5 ± 3.8 years. As shown on Table 1, after age stratification, 189 (29.3%) women were aged ≤ 35, 263 (40.8%) women were aged 36–39 years, and 193 (29.9%) women were aged ≥ 40 years. Based on the Bologna criteria [26], 124 (19.2%) of women were poor responders, and most of them were ≥ 40 years old (*P* < 0.001). No significant differences in ovarian diseases or surgeries were observed (Table 1).

**Table 1:** Demographic data and parameters of the oocyte freezing cycles

	Total	Age ≤ 35 years	Age 36–39 years	Age ≥ 40 years	*P* value
Cases/Cycles, n/N	645/840	189/221	263/324	193/295	
Two or more retrieval cycles	126 (19.5)	28 (14.8)	44 (16.7)	54 (28.0)	0.005
Single status	596 (92.4)	175 (92.6)	245 (93.2)	176 (91.2)	0.731
Childless	626 (97.1)	186 (98.4)	254 (96.6)	186 (96.4)	0.803
Body mass index, kg/m^2^	20.3 (19.0–21.9)	20.0 (18.7–21.5)	20.3 (18.9–21.8)	21.7 (19.2–22.5)	0.136
Bologna criteria for POR	124 (19.2)	25 (13.2)	29 (11.0)	70 (36.3)	< 0.001
**Ovarian disease**					
Endometriosis	53 (8.2)	22 (11.6)	13 (4.9)	18 (9.3)	0.088
Dermoid cyst	11 (1.7)	7 (3.7)	3 (1.1)	1 (0.5)	0.105
Other benign ovarian tumors	12 (1.9)	5 (2.6)	4 (1.5)	3 (1.6)	0.952
Prior ovarian surgeries	38 (5.9)	18 (9.5)	10 (3.8)	10 (5.2)	0.099
**Oocyte retrieval and freezing**					
Retrieved oocytes/case	12 (7–18)	16 (10–21)	13 (8–20)	9 (5–14)	< 0.001
Retrieved MII oocytes/case	8 (4–14)	11 (6–16)	9 (5–15)	6 (3–10)	< 0.001
Retrieved MII/total oocytes, %	73.9 (58.8–87.2)	75.0 (62.5–87.0)	75.0 (60.0–87.5)	71.0 (55.0–87.5)	0.396
Frozen oocytes/case	11 (7–17)	15 (9–20)	12 (7–19)	8 (4–13)	< 0.001
Frozen MII oocytes/case	9 (5–14)	12 (7–17)	10 (6–16)	6 (3–10)	< 0.001

Unless otherwise indicated, data are presented as the median number (IQR) or as the number/total number (n/N) (percentage). A *P*-value lower than 0.05 is defined as significantly different

*POR* poor ovarian response, *IQR* interquartile range, *MII* metaphase II

The relationship status was single in 596 women, which accounted for 92.4% of those who came for oocyte freezing cycles (Table 1). Among the other 49 (7.6%) women who were married, the reasons for oocyte freezing included unlicensed marriage status, unstable relationship, and disagreement between couples regarding IVF. Most women were childless at the time of oocyte freezing (Table 1). None of the aforementioned characteristics were significantly different among the three age groups. At the time of thawing oocytes, all women were married, while 43 (79.6%) women were single and 11 (20.4%) women were married when they had the oocytes frozen.

### Parameters of oocyte freezing cycles

The GnRH antagonist protocol (533 cycles, 63.5%) was mostly used for controlled ovarian stimulation, followed by the short GnRH agonist protocol (182 cycles, 21.7%) and the PPOS protocol (113 cycles, 13.5%). The average length of stimulation (presented as the mean ± standard deviation) was 10.0 ± 2.2 days. A total of 9033 oocytes were retrieved and 8344 oocytes (7017 MII oocytes) were frozen in the 840 cycles. The median number of total retrieved oocytes was 12 (IQR, 7–18; range, 0–62). The median percentage of matured oocytes was 73.9% (IQR, 58.8%–87.2%). The median number of total frozen MII oocytes was 9 (IQR, 5–14; range, 0–53) (Table 1). Eleven (1.3%) cycles occurred with no frozen oocytes and 3 (0.5%) patients did not freeze any oocyte. The freezing methods included slow freezing (28 cycles, 3.4%) and vitrification (796 cycles, 96.0%). In 5 (0.6%) cycles, one-half of the oocytes were slow frozen and one-half of them were vitrified. 

On comparing the three age groups, the group aged ≥ 40 years had the least number of oocytes retrieved and frozen (*P* < 0.001) and the highest percentage of women who had two or more retrieval cycles to accumulate oocytes (*P* = 0.005). The percentage of matured oocytes upon retrieval did not differ among the three groups (Table 1).

### Oocyte disposition, storage duration, and the usage rate

Fifty-four (8.4%) women thawed their oocytes, 9 (1.4%) women decided to discard them, and 15 (2.3%) women transported their oocytes to other infertility centers or abroad. Most of the women still preserved their oocytes, which accounted for 87.9% of cases. The median of storage duration was 3.0 (IQR, 1.4–4.7) years for women who thawed oocytes at our center and 3.5 (IQR 2.3–6.7) years for women who transported them outside our center. The median of storage duration was 9.5 (IQR, 6.7–12.1) years for women who discarded their oocytes, which was significantly longer than that of the other two dispositions (*P* = 0.008). 

The 15 women who transported their oocytes were not included in the calculation of usage rate because the actual utility of the oocytes and the pregnancy outcomes could not be followed up. Seven women transported their oocytes to other countries: one woman chose surrogacy, another woman opted for sperm donation, and five women were lost to follow-up. 

**Fig. 1:**
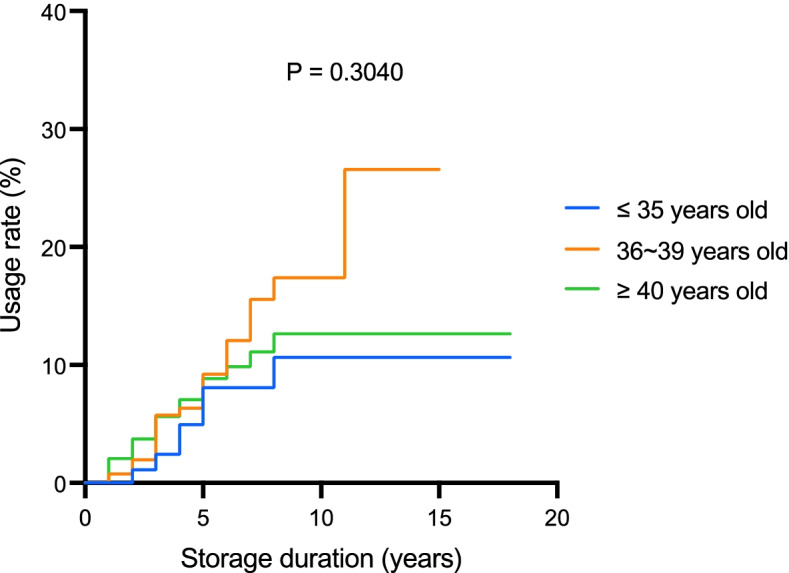
The probabilities of usage after storage years among the different age groups at oocyte freezing. The probability of each group was presented as a cumulative incidence curve. The probabilities of thawing the oocytes were 10.6%, 26.6%, and 12.7% for women who cryopreserved their oocytes at age ≤ 35 years, 36–39 years, and ≥ 40 years, respectively. No significant difference exists (*P* = 0.3040)

**Fig. 2:**
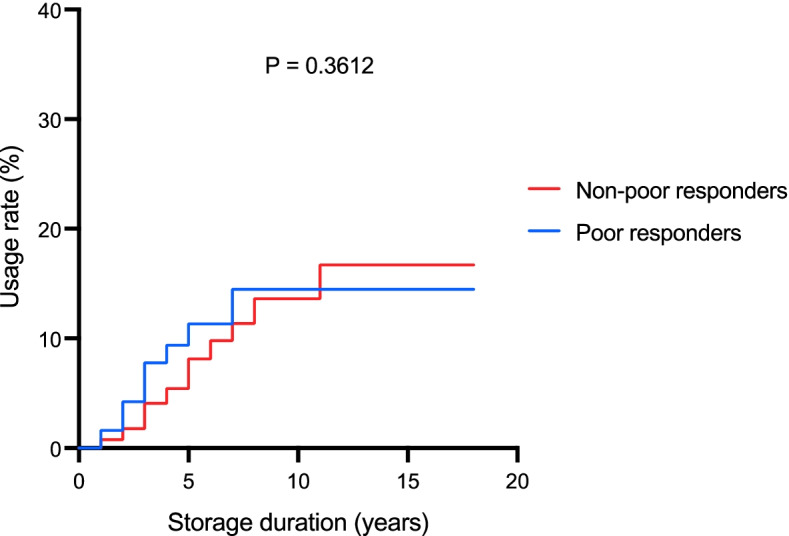
The probabilities of usage after storage years among the poor responder groups, stratified by the Bologna criteria. The probability of each group was presented as a cumulative incidence curve. No significant difference exists between the poor responder group and the non-poor responder group (*P* = 0.3612)

Among the 54 women who thawed their oocytes, the overall usage rate and storage duration were not significantly different among the three age groups (*P* = 0.650 and *P* = 0.817, respectively) (Table 2). The probability of thawing oocytes after years of storage was evaluated by using the cumulative incidence curve. As depicted in Fig. 1, after the storage duration exceeded 10 years, the probabilities of thawing the oocytes were 10.6%, 26.6%, and 12.7% for women aged ≤ 35 years, 36–39 years, and ≥ 40 years, respectively, who cryopreserved their oocytes. The curves of cumulative incidence across the three age groups showed no significant difference (*P* = 0.3040) (Fig. 1). No significant difference existed in the cumulative incidence rate between the poor responder group and the non-poor responder group (*P* = 0.3612) (Fig. 2).

**Table 2:** Parameters of the oocyte thawing cycles

	Total	Age ≤ 35 years	Age 36–39 years	Age ≥ 40 years	*P* value
Thawing cases	54	11	26	17	
Embryo transfer cases/cycles, n/N	41/52	11/16	21/26	9/10	
Low ovarian reserve at freezing cycles	11/54 (20.4)	0/11 (0.0)	3/26 (11.5)	8/17 (47.1)	0.009
Usage rate	54/645 (8.4)	11/189 (5.8)	26/263 (9.9)	17/193 (8.8)	0.650
Storage duration, y	3.0 (1.4–4.7)	3.4 (2.6–4.3)	3.1 (2.2–5.2)	2.8 (1.2–4.3)	0.817
Frozen oocytes/case	10 (5–15)	14 (10–18)	10 (7–15)	4 (2–10)	0.010
Thawed oocytes/case	9 (5–13)	13 (8–16)	9 (7–13)	4 (2–9)	0.017
Survived oocytes/case	7 (3–10)	10 (3–12)	8 (4–11)	3 (2–7)	0.054
Survival rate of all oocytes	382/518 (73.7)	100/147 (68.0)	205/262 (78.2)	77/109 (70.6)	0.159
Frozen MII oocytes/case	7 (5–13)	11 (6–17)	8 (6–13)	2 (2–10)	0.022
Thawed MII oocytes/case	7 (5–10)	8 (5–14)	8 (5–10)	2 (2–7)	0.033
Survived MII oocytes/case	5 (2–9)	7 (3–11)	5.5 (3–8)	2 (1–5)	0.077
Survival rate of MII oocytes	311/405 (76.8)	80/110 (72.7)	164/204 (80.4)	67/91 (73.6)	0.528
Fertilization rate	220/332 (66.3)	61/85 (71.8)	110/177 (62.1)	49/70 (70.0)	0.545
Cancel rate	13/54 (24.1)	0/11 (0.0)	5/26 (19.2)	8/17 (47.1)	0.038
Implantation rate	29/122 (23.8)	9/34 (26.5)	16/66 (24.2)	4/22 (18.2)	0.988
Clinical pregnancy rate/transfer	21/52 (40.4)	8/16 (50.0)	9/26 (34.6)	4/10 (40.0)	0.943
Ongoing pregnancy rate/transfer	18/52 (34.6)	7/16 (43.8)	8/26 (30.8)	3/10 (30.0)	0.958
Total deliveries	18	7 ^a^	8	3	
Total live births	21	7	11	3	
At least one live birth/thawed case	17/54 (31.5)	6/11 (54.5)	8/26 (30.8)	3/17 (17.6)	0.320
Cumulative live birth/thawed case	21/54 (38.9)	7/11 (63.6)	11/26 (42.3)	3/17 (17.6)	0.045
Live births/thawed MII oocytes	21/405 (5.2)	7/110 (6.4)	11/204 (5.4)	3/91 (3.3)	0.941

^a^One woman had two deliveries with one live birth each time

Unless otherwise indicated, data are presented as the median number (IQR) or as the number/total number (n/N) (percentage). A *P*-value lower than 0.05 is defined as significantly different

*IQR* interquartile range, *MII* metaphase II

### Oocyte thawing cycles and pregnancy outcomes

Among the 54 patients who came for oocyte thawing cycles, seven patients had more than one oocyte freezing cycle. In these seven participants, five cases decided to thaw all frozen oocytes in one cycle, one case had two oocyte thawing cycles and the other one thawed a part of oocytes in one cycle. Overall, 43 (79.6%) patients thawed all frozen oocytes concurrently for IVF and 3 (5.6%) patients received two oocytes warming cycles. The remaining 8 cases (14.8%) did not thawed all frozen oocytes at last, while five of them had live births after using a part of the cryopreserved oocytes, two of them did not return for a second thawing cycle after they failed to get pregnant in the first embryo transfer, and one of them transported the remaining oocytes abroad after no blastocyst formation for preimplantation genetic testing for aneuploidy (PGT-A) in the first oocyte thawing cycle.

As shown on Table 2, the survival rate of all oocytes was 73.7% and that of MII oocytes was 76.8%. The fertilization rate after ICSI was 66.3%. Overall, 75 MI oocytes were thawed; of these, 52 survived (i.e., survival rate, 72.2%). Twenty-one oocytes reached MII before fertilization. For these MII oocytes, the fertilization rate was 52.4%; the embryos were either not transferred due to poor quality or did not achieve pregnancy after embryo transfer. If MI oocytes were excluded, the fertilization rate would be 67.2% for the surviving MII oocytes. The survival rate and fertilization rate of MII oocytes were not significantly different across the three age groups (*P* = 0.528 and *P* = 0.545, respectively). The group aged ≥ 40 years had the least number of thawed and survived MII oocytes (*P* = 0.033 and *P* = 0.077, respectively). 

Two women opted for PGT-A; the outcome of one woman revealed no blastocyst formation for embryo biopsy, and the PGT-A report of the other woman showed no euploid embryo for transfer. Eight women underwent a fresh stimulating cycle to accumulate more embryos, which were excluded from the analysis of pregnancy outcomes after embryo transfer (Supplementary Fig. 2). The cancel rates of embryo transfer from the frozen oocytes were 0.0%, 19.2% and 47.1% (*P* = 0.038) in the groups aged ≤ 35 years, 36–39 years, and ≥ 40 years, respectively (Table 2). 

Total 122 embryos were transferred in 41 women with 52 cycles. Blastocyst transfer was achieved in 13.5% (7/52) cycles, and 86.5% (45/52) of embryo transfer cycles were transferred in the cleavage stage. The overall implantation rate was 23.8% (29/122). The clinical pregnancy rate was 40.4% (21/52) and the ongoing pregnancy rate was 34.6% (18/52) per transfer cycle. Seventeen women had live births, which accounted for 31.5% of cases who returned and thawed their oocytes. Twenty-one termed babies were delivered, which included three pairs of twins. The mean birth body weight was 3020 g. One woman had two deliveries with one live birth each time. Among women who cryopreserved their oocytes before 35 years of age, 54.5% had at least one live birth. At least one live birth in the other two groups occurred in 30.8% and 17.6% (*P* = 0.320) of individuals aged 36–39 years and ≥ 40 years, respectively. The cumulative live birth rate per thawed case were 63.6%, 42.3% and 17.6% (*P* = 0.045) in the groups aged ≤ 35 years, 36–39 years, and ≥ 40 years, respectively. The chance of live birth for each MII oocyte cryopreserved was 5.2% in overall; the chances of live birth for each MII oocytes were 6.4%, 5.4%, and 3.3% for those who cryopreserved oocytes at age ≤ 35 years, 36–39 years, and ≥ 40 years, respectively. 

In a multiple logistic regression model controlling for age at first oocyte freezing, number of thawed MII oocytes and storage years, only the number of thawed MII oocytes was predictive of live birth (B = 0.35; adjusted odds ratio [aOR], 1.42; 95% confidence interval [CI], 1.14–1.77; *P* = 0.002). The age at first oocyte freezing (B = -0.05; aOR, 0.95; 95% CI 0.72–1.25; *P* = 0.727) and the storage years (B = 0.24; aOR, 1.27; 95% CI 0.88–1.83; *P* = 0.205) were non-predictive of live births in our model.

### Cumulative costs and cost-effectiveness

The costs for oocyte freezing cycles and oocyte thawing cycles are presented in Table 3. The median of total costs for one woman starting from oocyte freezing to oocyte thawing and including annual preservation fee were (in United States dollars [USD]) $7,444, $7,271, and $6,273 (*P* = 0.067) for groups who cryopreserved oocytes at age ≤ 35 years, 36–39 years, and ≥ 40 years, respectively. However, the cumulative live birth rate per thawed case also decreased with age. Thus, the cumulative costs for one live birth, also defined as cost-effectiveness in our study, were USD $11,704, $17,189, and $35,642 (*P* < 0.001) for age groups ≤ 35 years, 36–39 years, and ≥ 40 years, respectively. In comparison with their older counterparts, cryopreservation of oocytes at a younger age was more cost-effective.

**Table 3:** Cumulative costs and cost-effectiveness analysis

	Total	Age ≤ 35 years	Age 36–39 years	Age ≥ 40 years	*P* value
Freezing cases	645	189	263	193	
Thawing cases	54	11	26	17	
Embryo transfer cases	41	11	21	9	
Delivery cases	17	6	8	3	
Total live births	21	7	11	3	
Cumulative live birth/thawed case	21/54 (38.9)	7/11 (63.6)	11/26 (42.3)	3/17 (17.6)	0.045
Oocyte freezing cycles per case, mean ± SD	1.28 ± 0.25	1.09 ± 0.20	1.46 ± 0.51	1.11 ± 0.17	0.608
Storage duration, y	3.0 (1.4–4.7)	3.4 (2.6–4.3)	3.1 (2.2–5.2)	2.8 (1.2–4.3)	0.817
Cost for oocyte freezing/cycle, USD	$3131 ($2843-$3404)	$3264 ($3092-$3474)	$3213 ($2864-$3442)	$2903 ($2681-$3066)	0.187
Cost for oocyte freezing/case, USD	$3223 ($2903-$3474)	$3343 ($3217-$3490)	$3237 ($2970-$3580)	$2951 ($2806-$3346)	0.202
Cost for oocyte thawing/cycle, USD	$1873 ($1152-$2127)	$1894 ($1498-$2502)	$1987 ($1384-$2188)	$1855 ($743-$1890)	0.474
Cost for oocyte thawing/case, USD	$2101 ($1855-$3177)	$3044 ($1899-$3738)	$2125 ($1873-$3177)	$1856 ($1011-$1922)	0.015
Cumulative cost/case, USD	$6905 ($5916-$8471)	$7444 ($6603-$9062)	$7271 ($6021-$8500)	$6273 ($5089-$6965)	0.067
Cumulative costs for one live birth, USD	$17,750	$11,704	$17,189	$35,642	< 0.001

USD to New Taiwan dollar was approximately 1:28 (retrieved on Mar 1^st^, 2022)

Unless otherwise indicated, data are presented as the median number (IQR) or as the number/total number (n/N) (percentage). A *P*-value lower than 0.05 is defined as significantly different

*SD* Standard deviation, *IQR* Interquartile range, *USD* United States dollar

### Subgroup analysis

For three women, 33 oocytes underwent slow freezing. No difference existed in the survival and fertilization rate between the oocytes that were slow frozen and those that were vitrified (Supplementary Table 3). After excluding the three cases whose oocytes were slowly frozen, the remaining 51 women had their oocytes vitrified and returned to warm the oocytes later. As shown on Supplementary Table 4, among those who cryopreserved oocytes at age ≤ 35 years, 36–39 years, and ≥ 40 years, 60.0%, 29.2%, and 17.6% (*P* = 0.193) of the thawed cases achieved at least one live birth. The cumulative live birth rates per thawed case were 70.0%, 41.7%, and 17.6% (*P* = 0.025) from the group aged ≤ 35 years, 36–39 years, and ≥ 40 years, respectively. The cumulative costs for one live birth were USD $10,285, $17,436, and $35,642 (*P* < 0.001) in groups aged ≤ 35 years, 36–39 years, and ≥ 40 years, respectively. 

Among the 54 cases underwent oocyte warming cycles, 11 women were poor responders at the time of oocyte freezing. The distribution of age group was three cases between 36–39 years and eight cases ≥ 40 years. The accumulated oocytes were 7, 14 (in six oocyte retrieval cycles), and 24 (in five oocyte retrieval cycles) for the three cases aged between 36–39 years. For the eight cases ≥ 40 years, the number of accumulated oocytes ranged from 1 to 4. There was no live birth from the thawed oocytes in this subgroup of poor responders. For the poor responders, the cost to achieve a live birth could be higher in order to collect more oocytes. However, the expenditure for one live birth could not be estimated based on our present data.

## Discussion

In this cohort, we explored the dispositions of planned cryopreserved oocytes and their clinical outcomes after thawing cycles in our center. We found that with no option for sperm donation for unmarried individuals, the overall usage rate of the oocytes was 8.4%. The probability of thawing the oocytes was higher after a longer storage duration for women who underwent oocyte cryopreservation between 36 and 39 years of age. Among those who opted to thaw their oocytes, 31.5% of cases experienced live births. However, the chance of having at least one live birth decreased by age, which were 54.5%, 30.8%, and 17.6% for individuals who cryopreserved at age ≤ 35 years, 36–39 years, and ≥ 40 years, respectively. The cumulative costs for one live birth dramatically increased from USD $11,704 to $17,189 to $35,642 for women who cryopreserved oocytes at age ≤ 35 years, 36–39 years, and ≥ 40 years, respectively. In this study, the actual costs from oocyte freezing to oocyte thawing and the live birth rate from the thawed oocytes were calculated case by case, which we consider to be a strength of our study. Our study also provided the usage rate with real-world data in the situation in which sperm donation was inaccessible to unmarried women. The results presented in this paper are significant for the future studies concerning planned oocyte cryopreservation and cost-effectiveness analysis. 

As is well known, freezing the oocytes at a younger age and accumulation of more mature oocytes result in a better success rate of live birth, which was proven in our study and in other previous studies [12–15, 27]. A decreased percentage of euploid embryos has been reported among individuals with the intent of PGT-A, which were 51.7%, 32.4%, 31.3%, and 0% of women who cryopreserved their oocytes at the age < 35 years, 35–37 years, 38–40 years, and ≥ 41 years of age, respectively [14]. The association between the aneuploidy rate and advanced maternal age could not be evaluated in our study because very few individuals underwent PGT-A. The cumulative live birth rate per thawed case and the probability of cancellation both reflected a decreased success rate after aging in our study (Table 2). However, we did not detect a predictive value on age at oocyte freezing in the multiple logistic regression model, which may be confounded by the more significant factor of the number of thawed oocytes in our study. 

Our study uncovered a relatively low usage rate overall and after a longer duration of storage (Table 2 and Fig. 1). To date, data on the largest cohort done by Cobo et al. [12, 15] reported the usage rate between 9.3%–12.1%. A higher usage rate was presented from a Swedish center [13], which was 15%. Another United States-study [14] found a usage rate of 38.1% after 10–15 years of observation after oocyte freezing. The usage rate of each age group ranged from 25.0% to 44.1%; the group with the highest usage rate were women who had their oocyte frozen during the ages of 35–37 years. In our study, we noticed that the overall usage rate (i.e., 8.4%) was lower than the usage rate reported by Cobo et al. (i.e., 12.1%) [12] and Wennberg et al. (i.e., 15%) [13]. If a longer duration of observation was considered, the highest usage rate may have only reached approximately 26.6% after 10 years of storage for individuals who cryopreserved their oocytes at the age of 36–39 years, which was also lower than the usage rate reported by Blakemore et al. [14]. 

The value of elective oocyte freezing remains under debate [8–11]. However, to the best of our knowledge, the previous cost-effectiveness analyses were based on assumed models instead of real-world data. The estimated cost for one live birth was USD $13,990 (€12,326, whereas the exchange rate was 1:1.14) in a Netherland-based study [8] and USD $39,946 in a United States-based study [11]. The aforementioned two studies contradicted the advantages of oocyte freezing. However, both teams agreed that the huge difference was likely originated from the different assumptions of age at oocyte freezing, which was 25 years old in the United States study and 35 years old in the Netherland study. Also, the costs were widely varied between the two groups. To evaluate the age that was most cost-effective for oocyte cryopreservation, a German-based study [10] reported a cost of USD $27,837, $25,445, and $29,045 USD (€24,526, €22,418, and €25,590) per live birth if oocyte cryopreservation was conducted at the age of 25 years, 30 years, and 38 years, respectively. In our study, we calculated the actual cost per live birth, based on the charges of each medication, the procedure, and the chances of live birth at our infertility center. The cumulative cost for one live birth was doubled in the group aged ≥ 40 years than that for the group aged 36–39 years, and the cost was threefold higher in the group aged ≥ 40 years than that in the group aged ≤ 35 years. 

A previous review article [28] has implied that the use of cryopreserved oocytes is too low to show the benefits of elective oocyte freezing. Oocyte freezing can be cost-effective only if its usage rate reaches 50% and the use of oocytes is not restricted to heterosexual married couples. Two previous studies from Spain [15] and the United States [14] interestingly reported that 41.7% and 37.5% of individuals, respectively, opted to use donor sperm. However, donor sperm is not accessible to unmarried women in Taiwan [16]. The last resort for such women could be transporting the oocytes abroad to seek donor sperm, whereas this option causes additional financial burden. We believe that the regulations limit the usage of oocytes for single women in our society and prolong the decision of disposition if they are not in a stable relationship.

The ultra-low fertility rate in the past decades has raised public concerns in Taiwan. Based on the official statistics, in the past 10 years, the mean age at first marriage has increased by almost 2 years and the mean age at first delivery has increased by 3 years [29, 30]. Several factors that contribute to the low fertility rate include delayed marriage or even being single for life [31]. Industrialization, expansion of tertiary education, and an increased female labor force have been proposed as reasons for delayed marriage [31]. Younger women may opt to pursue higher education or a career over marriage or childbearing. In addition, low acceptance of nonmarital birth in the East Asian society is another negative factor that hinders women from becoming pregnant, owing to instability in their relationships. The community is becoming more open-minded; however, childbearing is usually not an option for unmarried women [32]. 

To preserve fertility, oocyte cryopreservation is a reasonable option for unmarried women who wish to defer childbearing before self-accomplishment. Although planned oocyte cryopreservation gives women the hope of raising a biological child at a later age, the fact that a live birth is not guaranteed should be emphasized in a thorough consultation. The evidence of utilization (e.g. usage rate 8.4%), the pregnancy outcome of the cryopreserved oocytes (e.g. live birth rate) and the cost-effectiveness of oocyte freezing should be provided as a reference to those who asks for planned oocyte cryopreservation. The psychological and behavioral changes after oocyte freezing should also be foreseen. The fact that an older maternal age has the increased risk of pregnancy-related complications, which cannot be overcome by freezing oocytes at a younger age, should also be discussed during a consultation [1, 2]. 

The limitations of our study are as follows. First, although the number of oocyte freezing cases was sufficiently large to show the characteristics of our cohort, the clinical results remained limited because only a small proportion of cases returned and thawed oocytes in our center. Second, the extrapolation may be limited because of different cultural backgrounds regarding the utility of oocyte freezing and the costs for assisted reproduction techniques. Third, this study was a retrospective observational study. Approximately 13.7% of our cases were lost to follow-up for more than 2 years, despite being notified by text messages. Thus, we were unaware of their current status of marriage, reproductive experience, and willingness to preserve the unused oocytes. Additionally, the clinical outcomes after cases who transported the oocytes abroad were not completely followed up. The usage rate may be underestimated because we excluded these cases from the analysis of thawing cycles.

## Conclusion

The emergence of planned oocyte cryopreservation has been a reasonable choice for single women to preserve fertility in the absence of a suitable partner or if women simply wish to defer childbearing to achieve their professional goals. We found a low usage rate in our cohort. The cumulative live birth rate was greatest in the youngest group and the cumulative cost per live birth was highest in the oldest group, which was threefold greater than that in the group aged ≤ 35 years. This study also contributed a view of cost-effectiveness in the real-world, which would hopefully aid future analysis and decision-making for public health policy and women willing to preserve fertility.

## Supplementary Information

**Additional file 1: Supplementary Figure 1. **The trend of the social oocyte freezing cycle per year in our center gradually grew from 2002 to 2020 and significantly increased in the past 10 years (e.g., 2010–2020).

**Additional file 2: Supplementary Figure 2.** The flowchart of the cases underwent oocyte thawing and embryo transfer cycles from the cryopreserved oocytes. ET: embryo transfer; PGT-A: preimplantation genetic testing for aneuploidy.

**Additional file 3: Supplementary Table 3. **The survival, fertilization and delivery rate between the oocytes that were slow frozen and the oocytes that were vitrified.

**Additional file 4: Supplementary Table 4. **Live birth rate and cumulative costs of the thawed cases whose oocytes were vitrified. The cases whose oocytes were slow frozen at the time of oocyte freezing were excluded.

## Data Availability

The datasets used and analysed during the current study available from the corresponding author on reasonable request.

## Abbreviations

IVF: In-vitro fertilization
ESHRE: European Society for Human Reproduction and Embryology
IRB: Institutional Review Board
GnRH: Gonadotropin-releasing hormone
PPOS: Progestin-primed ovarian stimulation
MII: Metaphase II
MI: Metaphase I
PROH: 1,2-Propanediol
ICSI: Intracytoplasmic sperm injection
2PN: Two pronuclei
IQR: Interquartile range
PGT-A: Preimplantation genetic testing for aneuploidy
USD: United States dollars
ET: Embryo transfer
