# Supplementary figures and images for: Usage and cost-effectiveness of elective oocyte freezing: a retrospective observational study
Source: Reprod Biol Endocrinol. 2022 Aug 16;20:123. doi: 10.1186/s12958-022-00996-1 (PMC9380307; doi:10.1186/s12958-022-00996-1)

## Slide 1
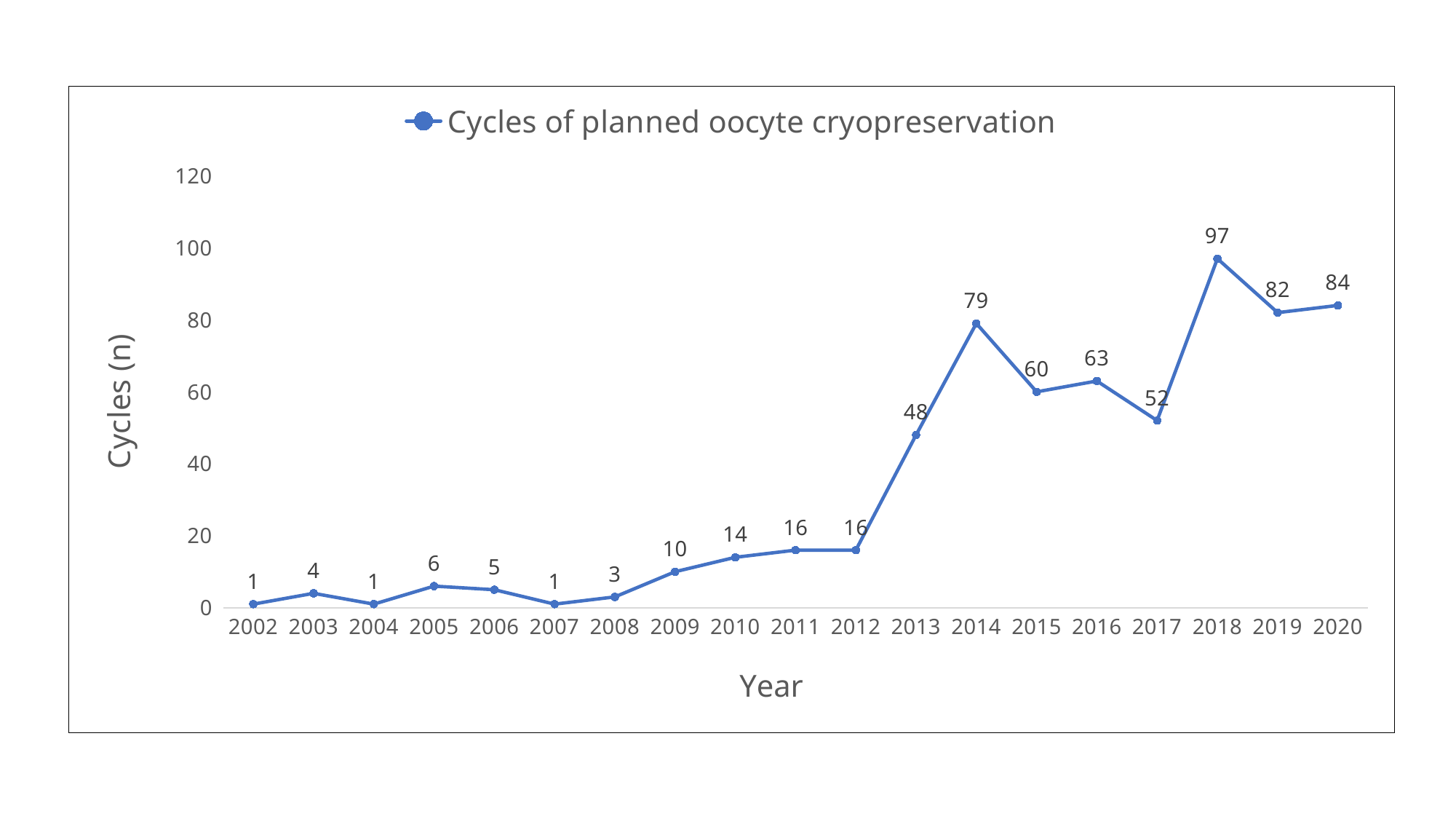

### Chart
| Category | Cycles of planned oocyte cryopreservation |
|---|---|
| 2002 | 1.0 |
| 2003 | 4.0 |
| 2004 | 1.0 |
| 2005 | 6.0 |
| 2006 | 5.0 |
| 2007 | 1.0 |
| 2008 | 3.0 |
| 2009 | 10.0 |
| 2010 | 14.0 |
| 2011 | 16.0 |
| 2012 | 16.0 |
| 2013 | 48.0 |
| 2014 | 79.0 |
| 2015 | 60.0 |
| 2016 | 63.0 |
| 2017 | 52.0 |
| 2018 | 97.0 |
| 2019 | 82.0 |
| 2020 | 84.0 |

Supplement: Supplementary file 1 — Additional file 1: Supplementary Figure 1. The trend of the social oocyte freezing cycle per year in our center gradually grew from 2002 to 2020 and significantly increased in the past 10 years (e.g., 2010–2020). [file 12958_2022_996_MOESM1_ESM.pptx]
