# Supplementary material for: Usage and cost-effectiveness of elective oocyte freezing: a retrospective observational study
Source: Reprod Biol Endocrinol. 2022 Aug 16;20:123. doi: 10.1186/s12958-022-00996-1 (PMC9380307; doi:10.1186/s12958-022-00996-1)
Supplement: Supplementary file 2 — Additional file 2: Supplementary Figure 2. The flowchart of the cases underwent oocyte thawing and embryo transfer cycles from the cryopreserved oocytes. ET: embryo transfer; PGT-A: preimplantation genetic testing for aneuploidy. [file 12958_2022_996_MOESM2_ESM.pptx]

## Slide 1
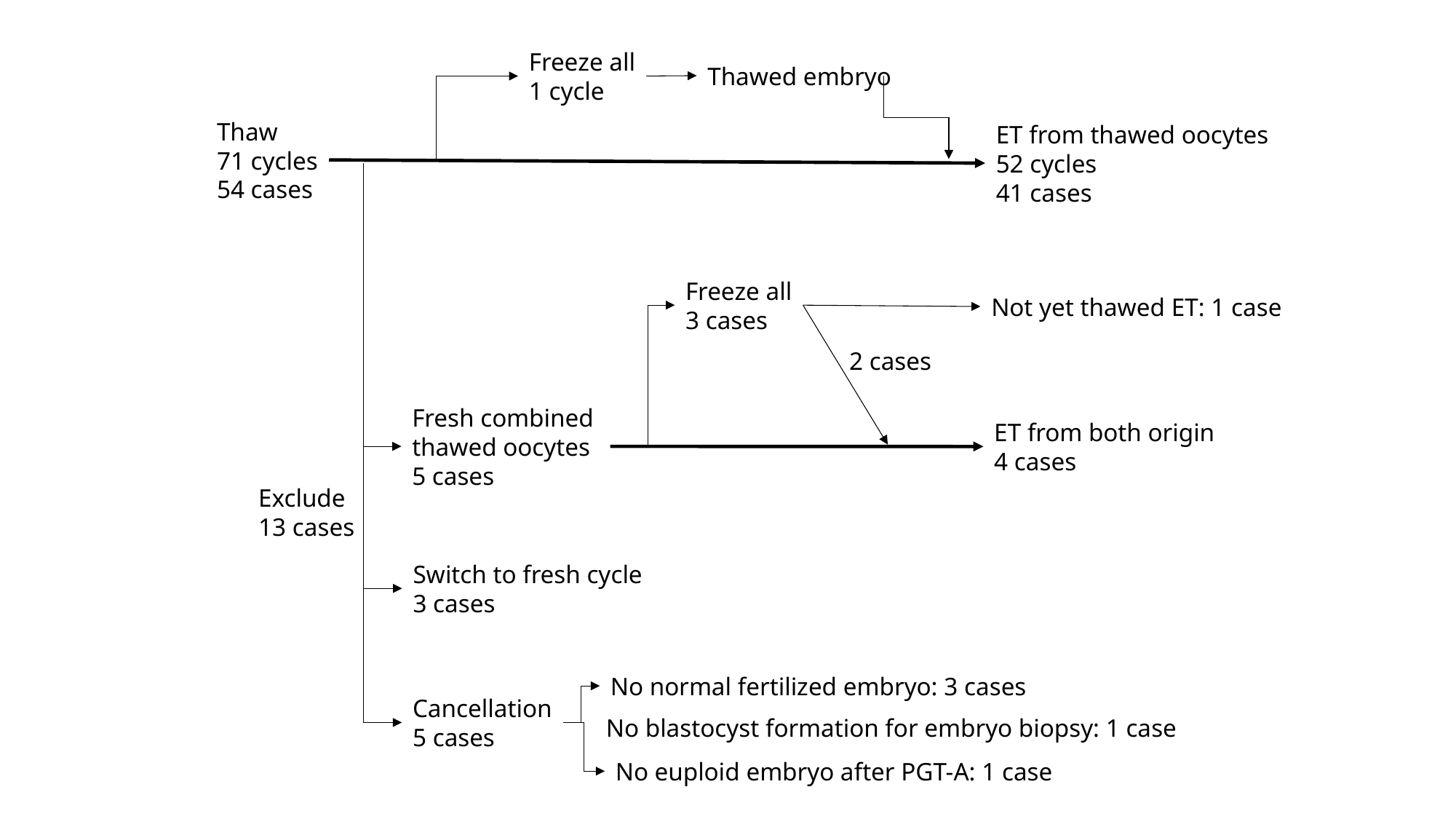

Freeze all
1 cycle
Thawed embryo
Thaw
71 cycles
54 cases
ET from thawed oocytes
52 cycles
41 cases
Freeze all
3 cases
Not yet thawed ET: 1 case
2 cases
Fresh combined thawed oocytes
5 cases
ET from both origin
4 cases
Exclude
13 cases
Switch to fresh cycle
3 cases
No normal fertilized embryo: 3 cases
Cancellation
5 cases
No blastocyst formation for embryo biopsy: 1 case
No euploid embryo after PGT-A: 1 case
