# Supplementary material for: Usage and cost-effectiveness of elective oocyte freezing: a retrospective observational study
Source: Reprod Biol Endocrinol. 2022 Aug 16;20:123. doi: 10.1186/s12958-022-00996-1 (PMC9380307; doi:10.1186/s12958-022-00996-1)
Supplement: Supplementary file 3 — Additional file 3: Supplementary Table 3. The survival, fertilization and delivery rate between the oocytes that were slow frozen and the oocytes that were vitrified. [file 12958_2022_996_MOESM3_ESM.docx]

**Supplementary Table 3.** The survival, fertilization and delivery rate between the oocytes that were slow frozen and the oocytes that were vitrified.

|  | Slow freezing | Vitrification | *P* value |
| --- | --- | --- | --- |
| Case number | 3 | 51 |  |
| Oocyte number | 33 | 485 |  |
| Survival rate of all oocytes | 24/33 (72.7) | 358/485 (73.8) | 0.891 |
| Survival rate of MII oocytes | 14/18 (77.8) | 297/387 (76.7) | 0.919 |
| Fertilization rate | 11/17 (64.7) | 209/315 (66.3) | 0.889 |
| Delivery cases | 1 (33.3) | 16 (31.4) | 0.943 |
| Total live births | 1 (33.3) | 20 (39.2) | 0.876 |

^a^ One woman had two deliveries with one live birth each time.

Unless otherwise indicated, data are presented as the number (percentage). A *P*-value lower than 0.05 is defined as significantly different.
