# Supplementary material for: Usage and cost-effectiveness of elective oocyte freezing: a retrospective observational study
Source: Reprod Biol Endocrinol. 2022 Aug 16;20:123. doi: 10.1186/s12958-022-00996-1 (PMC9380307; doi:10.1186/s12958-022-00996-1)
Supplement: Supplementary file 4 — Additional file 4: Supplementary Table 4. Live birth rate and cumulative costs of the thawed cases whose oocytes were vitrified. The cases whose oocytes were slow frozen at the time of oocyte freezing were excluded. [file 12958_2022_996_MOESM4_ESM.docx]

**Supplementary Table 4.** Live birth rate and cumulative costs of the thawed cases whose oocytes were vitrified. The cases whose oocytes were slow frozen at the time of oocyte freezing were excluded.

|  | Total | Age ≤35 years | Age 36–39 years | Age ≥40 years | *P* value |
| --- | --- | --- | --- | --- | --- |
| Freezing cases | 612 | 179 | 253 | 180 |  |
| Thawing cases | 51 | 10 | 24 | 17 |  |
| Embryo transfer cases | 39 | 10 | 20 | 9 |  |
| Delivery cases | 16 | 6 ^a^ | 7 | 3 |  |
| Total live births | 20 | 7 | 10 | 3 |  |
| At least one live birth/thawed case | 16/51 (31.4) | 6/10 (60.0) | 7/24 (29.2) | 3/17 (17.6) | 0.193 |
| Cumulative live birth/thawed case | 20/51 (39.2) | 7/10 (70.0) | 10/24 (41.7) | 3/17 (17.6) | 0.025 |
| Cumulative costs/case, USD | $6899 ($5593-$8311) | $7200 ($6603-$8311) | $7271 ($6021-$8500) | $6273 ($5089-$6965) | 0.037 |
| Cumulative costs for one live birth, USD | $17599 | $10285 | $17436 | $35642 | < 0.001 |

^a^ One woman had two deliveries with one live birth each time.

The preservation fee was approximately USD $303.00. The exchange rate of USD to New Taiwan dollar was approximately 1:28 (retrieved on Mar 1^st^, 2022).

Unless otherwise indicated, data are presented as the median number (IQR) or as the number/total number (n/N) (percentage). A *P*-value lower than 0.05 is defined as significantly different.

SD, standard deviation; IQR, interquartile range; USD, United States dollar.
